# On the use of aggregated human mobility data to estimate the reproduction number

**DOI:** 10.1038/s41598-021-02760-8

**Published:** 2021-12-02

**Authors:** Fabio Vanni, David Lambert, Luigi Palatella, Paolo Grigolini

**Affiliations:** 1 CNRS, GREDEG, Université Côte d’Azur, Nice, France; 2 Sciences Po, OFCE, Campus de Sophia Antipolis, Nice, France; 3 Center for Nonlinear Science, University of North Texas, Denton, TX USA; 4 Department of Mathematics, University of North Texas, Denton, TX USA; 5 Liceo Scientifico Statale “C. De Giorgi”, Lecce, Italy

**Keywords:** Statistical physics, Thermodynamics, Viral infection

## Abstract

The reproduction number of an infectious disease, such as CoViD-19, can be described through a modified version of the susceptible-infected-recovered (SIR) model with time-dependent contact rate, where mobility data are used as proxy of average movement trends and interpersonal distances. We introduce a theoretical framework to explain and predict changes in the reproduction number of SARS-CoV-2 in terms of aggregated individual mobility and interpersonal proximity (alongside other epidemiological and environmental variables) during and after the lockdown period. We use an infection-age structured model described by a renewal equation. The model predicts the evolution of the reproduction number up to a week ahead of well-established estimates used in the literature. We show how lockdown policies, via reduction of proximity and mobility, reduce the impact of CoViD-19 and mitigate the risk of disease resurgence. We validate our theoretical framework using data from Google, Voxel51, Unacast, The CoViD-19 Mobility Data Network, and Analisi Distribuzione Aiuti.

## Introduction

Understanding the effectiveness of public service announcements and large-scale physical distancing interventions is critical for managing the short and long-term phases of spread of the epidemic, as in the case of the CoViD-19 epidemic. Many countries have reacted via intervention strategies based on mobility and physical lockdowns together with regional and international border restrictions^1–4^. Many of these intervention policies are based on assessing the risk of an outbreak through compartmental disease models^5–9^. We intend our model to be complementary to other well-assessed estimates of the reproduction number. These estimates are based on phenomenological models which provide a starting point for estimation of key transmission parameters, such as the reproduction number, and forecasts of epidemic impact^10–14^.

From a practical point of view, it is fundamental to understand which approach best permits one to forecast epidemic dynamics in the presence of incomplete data. This is especially true when a country’s healthcare system is overwhelmed and data collection becomes sporadic. It is also important during the early phases of disease spread, when testing is incomplete or non-existent. For CoViD-19 there is the additional problem of undocumented cases^15,16^.

In our analysis, we focus our attention on the contribution of asymptomatic or undiagnosed (and thus undocumented) individuals to the propagation of the contagion, assuming that these hidden infectious agents have the ability to spread the disease in an environment where susceptible agents are present and all the individuals have uniform mobility and physical proximity parameters. Consequently, we evaluate the impact of physical distancing policies in response to the CoViD-19 epidemic in Italy, the US, and selected European locations. Our model is a renewal SIR model with a time-dependent contact rate. We provide an expression for the contact rate using real-world mobility and social distancing data from Google and other providers. Our approach is complementary to typical (fixed contact-rate, non-renewal) compartmental SIR models, with two essential differences: the time dependence of $\beta$ (contact rate in the SIR model) and the fact that we split $\beta$ into several factors.

In our model we formulate a specific factorization of the time-dependent contact rate into variables directly related to mobility and social distancing behaviors from real world data, together with other epidemiological and clinical variables.

In particular, we believe that the advantage of our model is that it is parsimonious in capturing the aggregated reproduction trends, splitting the contributions of different factors of a disease diffusion framework. Our model posits that undiagnosed individuals, captured by the variable $\lambda$, drive and sustain the infection process through a contact web disentangled into two aspects: mobility as movement trends and proximity as proxy for interpersonal distance. This framework takes into account a natural delay between time of contacts and the triggering of new infection chains, making the model a forward estimate for the values of $R_t$ to come. This perspective can provide useful insight for policy makers and regulators planning mobility restrictions or other strategies for mitigating the diffusion of an epidemic like CoViD-19.

We interpret this approach in terms of a macroscopic collision theory of infected individuals in a region with a given susceptible population, taking into account the mobility of individuals as well as their radii of interaction as reliable proxies of physical distancing measures (as explained in the “Methods” section):

1 $$\begin{aligned} R_{t}= {R}_0 {\tilde{S}}_t\tilde{\mathscr {B}}_t. \end{aligned}$$

Here, the tildes indicate that the variable is to be evaluated with a delay of $\tau _g$, a variable that accounts for the typical time it takes to observe newly generated positive tests (see^17,18^ and the “Calibration” section). ${\hat{R}}_0$ represents the reproduction number calculated in a given period of time which also embodies the constant contribution over that time. Next, $S_t$ is the fraction of individuals that are susceptible. Finally, ${\mathscr {B}}_{t}$ is the transmission rate function, which depends on average contact frequency, the virus’s infectiousness, and the infectious age of individuals in the contagion process. In this way, it is a generalization of the interaction variable in compartmental models.

The model of Eq. (1) is distinguished from other estimates in literature by its forward rather than backward-looking estimation procedure. We name the model proposed in this paper as the social distancing based model, aka SDM. Imagine that some infectious individuals have not been detected and isolated. We wish to evaluate a measure of risk of exposure for a given susceptible individual. We take a kinetic approach to the evaluation of this risk.

We imagine unobserved spreaders are free to infect other individuals and that the contagion acts within a certain radius *r* of an infected individual. We imagine an environment in which two types of individuals are present at a calendar time *t*. $n_s$ is the density of susceptible individuals in a region, while *j* is the density of actual new infected individuals where diagnosed and undocumented cases are both taken into consideration, as discussed in the “Methods” section. We consider the regional mobility, $\nu$, to be the average distance explored by each individual during the time interval, $\Delta t$, (usually daily). We define the distance, *r*, to be the maximum distance that an infected person can be from a susceptible person (in the model) and still cause them to become infected. This distance depends, for example, on the virus’ infectiousness as a function of distance and on the use of personal protective equipment, which can create a physical barrier so increasing effective distances. Physical distancing regulations, personal protection devices (such as mask wearing), and hygienic norms will result in a decrease in *r*, as also assessed in^19,20^. The interpersonal proximity $\rho$ has an inverse effect on infectivity to that of the interaction radius and it is defined to be the inverse of the square root of the average density of individuals in a region as discussed in the S.I. in more detail.

As specified in Table 1, $\lambda$ represents the efficiency of detecting real cases of infection, and takes on values between 0 and 1. In particular, $\lambda$ would have a value equal to 1 if testing and contact tracing technologies were maximally efficient, and its value approaches 0 if very few in a large infected population are detected, as shown by^16,21^. The value of $\lambda$ changes with infection age as well as *t* during the disease outbreak. These changes might depend, for example, on the ability to detect and isolate individuals, or the efficiency of contact tracing during the epidemic. Contact tracing efficiency varies with the characteristics of the infection and the speed and coverage of the tracing process.

Centralized manual testing and tracing may become an impractical strategy and a lockdown may become a more efficient and effective means of controlling an epidemic. However, lockdowns are not sustainable in the long term because of their social, economic, physical, and psychological effects. Lockdown policies have reduced the spread of CoViD-19, but as restrictions are relaxed transmission often goes up again.

Finally, the number of people at risk (susceptible individuals) is

$$\begin{aligned} n_s = n-\sum _{i=\tau _g}^\infty j(t-i) =n - \sum _{i=\tau _g}^\infty \frac{j_o(t-i) }{ \lambda (t-i)}, \end{aligned}$$

where *n* is initial fraction of susceptible population respect to the total population, and $j_0$ is the density of only the new diagnosed cases (official data) which is only a fraction of the total actual cases of new infections, note that $j_o(t)=j(t)=0$ for $t<0$. There are various factors which contribute to the transmission of a disease. The biological and environmental properties are accounted for in the transmissivity variable $\eta$, as explained in the “Methods” section. Physical proximity, viral load, and environmental conditions determine the infectious dose necessary to trigger the infection in a new host. For example, enclosed environments such as workplaces and schools correspond to higher $\eta$ values in the model as compared to an outdoor space. A summary of all the variables in the model appears in Table 1.

**Table 1:** Parameters of kinetic approach to infectious contacts.

Collision variable	Description
Mobility $\nu$	Movement trends over time
Social movements	Average speed of individual movements, can include distanced traveled per day and mobility trends
Contact zone *r*	The radius within which contact with an infectious individual can trigger a secondary infection in airborne diseases (infectious cross section)
Physical proximity $\rho$	The average effective distance between individuals for an airborne disease, a function of physical distance, protection devices, and hygienic procedures
Transmissibility $\eta$	The chance that a contact will result in an infection
Virus-host-environment interaction	Infectiousness due to environmental conditions as well as the virus’s ability to be more or less contagious. (Virus strain mutations, viral load, shedding, and immune system response are involved. Air flow, UV exposure, climate factors such as temperature and humidity that influence infectiousness )
Test and trace $\lambda$	Ability to detect and isolate contagious individuals
Testing efficacy and contact tracing	Analyzing samples to assess the current or past presence of SARS-CoV-2 viral (molecular and antigen) tests and antibody test. Identification of persons who may have come into contact with an infected person

Now let us recall the actual (or effective) reproduction number which represents the average number of secondary infections generated by each new infectious case (assuming $n_s$ and other environmental variables retain their current values forever). The actual reproduction number can be used as a predictive tool to track the epidemic’s evolution. It is also a measure of epidemic risk, in the sense that if it is significantly above one for long enough, then an outbreak will occur. Thus, by linking a dynamical model with time-series data, one obtains a measure of epidemic risk. This risk is derived (see “Methods” section) leading to the effective reproduction number:

2 $$\begin{aligned} {R(t)}\sim R(t_0) \dfrac{n_s(t-\tau _g)}{n_s(t_0-\tau _g)} \dfrac{\rho (t_0-\tau _g)}{\rho (t-\tau _g)} \dfrac{\nu (t-\tau _g)}{\nu (t_0-\tau _g)} , \end{aligned}$$

where $t_0$ is an initial (or calibration) time, and we have taken the testing efficiency $\lambda$ and the transmissibility $\eta$ constant during the lockdown periods, as discussed in the “Methods” section. We call the reproduction number as expressed in the previous equation $R_t$ SDM.

## Results

The above equation represents the change in the average number of secondary cases caused by a single primary case throughout the course of infection at calendar time *t* calibrated at an initial value (for example, before the lockdown). In the present section, we apply Eq. (2) to data from various sources in order to validate our modeling framework.

We assume the spatial homogeneity of every variable. In particular, $\rho$ to be the average proximity between individuals, and $\nu (t)$ to be their average mobility. Moreover we consider the fraction of missed cases, $\lambda$, to be constant with respect to infection age. Additionally, we define a typical time interval, the generative time $\tau _g$ as the average infection age at which positive test result is generated.

**Figure 1:**
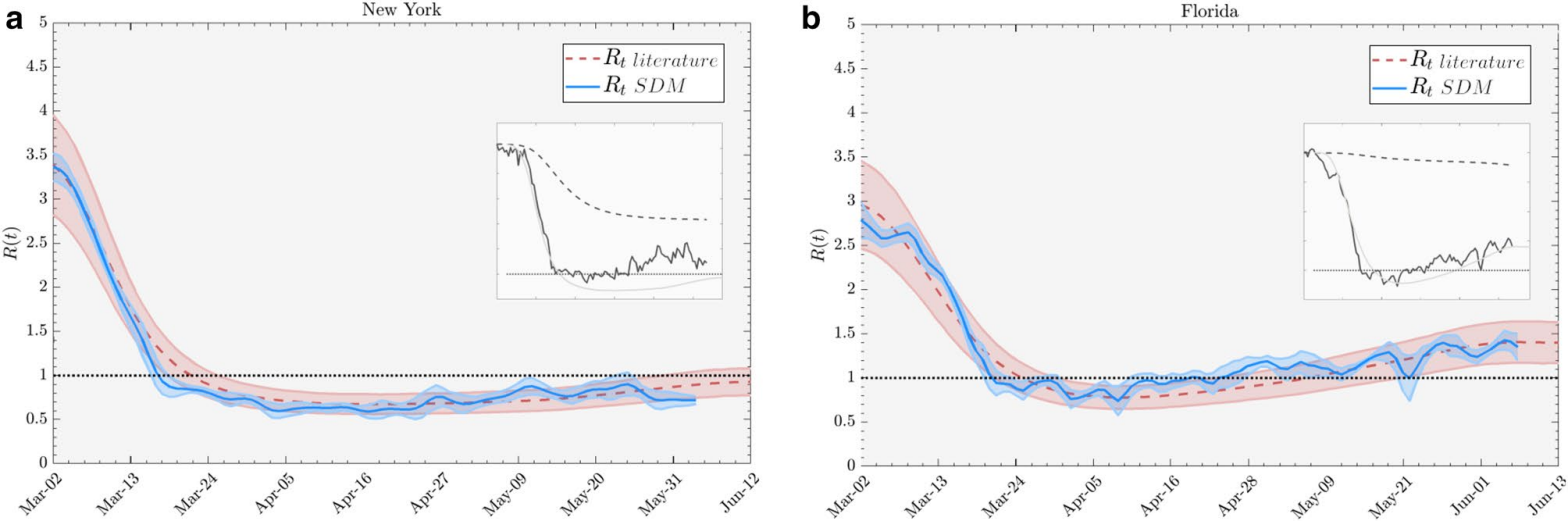
Reproduction number estimates for two US states. Comparison between the reproduction number calculated from symptom onset data as in literature^22^ (dashed red line) and the reproduction number computed according our kinetic SDM approach, using data from^23^ for mobility^24^, for social proximity and^25^ for epidemic data. Ribbons are the $90\%$ credible interval obtained via bootstrapping. Insets represent the single components of the reproduction number as in Eq. (1), specifically solid black and gray line is *R*(*t*) using only mobility and interpersonal proximity variables respectively, and dashed black line is *R*(*t*) due to the depletion of susceptibles only. The scale of the insets are the same the main plot. Calibration coefficients in the two examples are $c=1.21$ and $c=1.05$ respectively (see “Calibration” subsection of Methods).

The changing trends of the reproduction number may be due to several interrelated reasons apart from physical distancing policies. These reasons can be collected into two groups. The first has to do with the virus itself and its capacity to spread. Favorable environmental conditions or the emergence of less dangerous strains can decrease the effective infectiousness of the contagion. The other group of reasons is connected to the decrease in the susceptible population. On the other hand, physical distancing (also known as social distancing) is a practice recommended by public health officials to stop or slow down the spread of contagious diseases. It requires maintaining physical space between individuals who may spread certain infectious diseases. The data repositories used to obtain our results are listed in the Supplementary Information.

As proxies for mobility we consider both the changes in movement fluxes and percent change in average distance traveled, as released respectively in Google’s mobility report^23^ and Unacast’s scoreboard^26^. We take the mobility to represent the average relative speed of the individuals with respect to each other. The fact that we use *relative* velocity is important, as it properly accounts for situations in which people move rapidly in a coordinated way.

We infer a measure of proximity from the active population density, i.e., the number of people per unit area moving about in selected locations, as variously reported by Voxel51’s proximity index^24^ and Unacast’s human encounters^26^.

**Figure 2:**
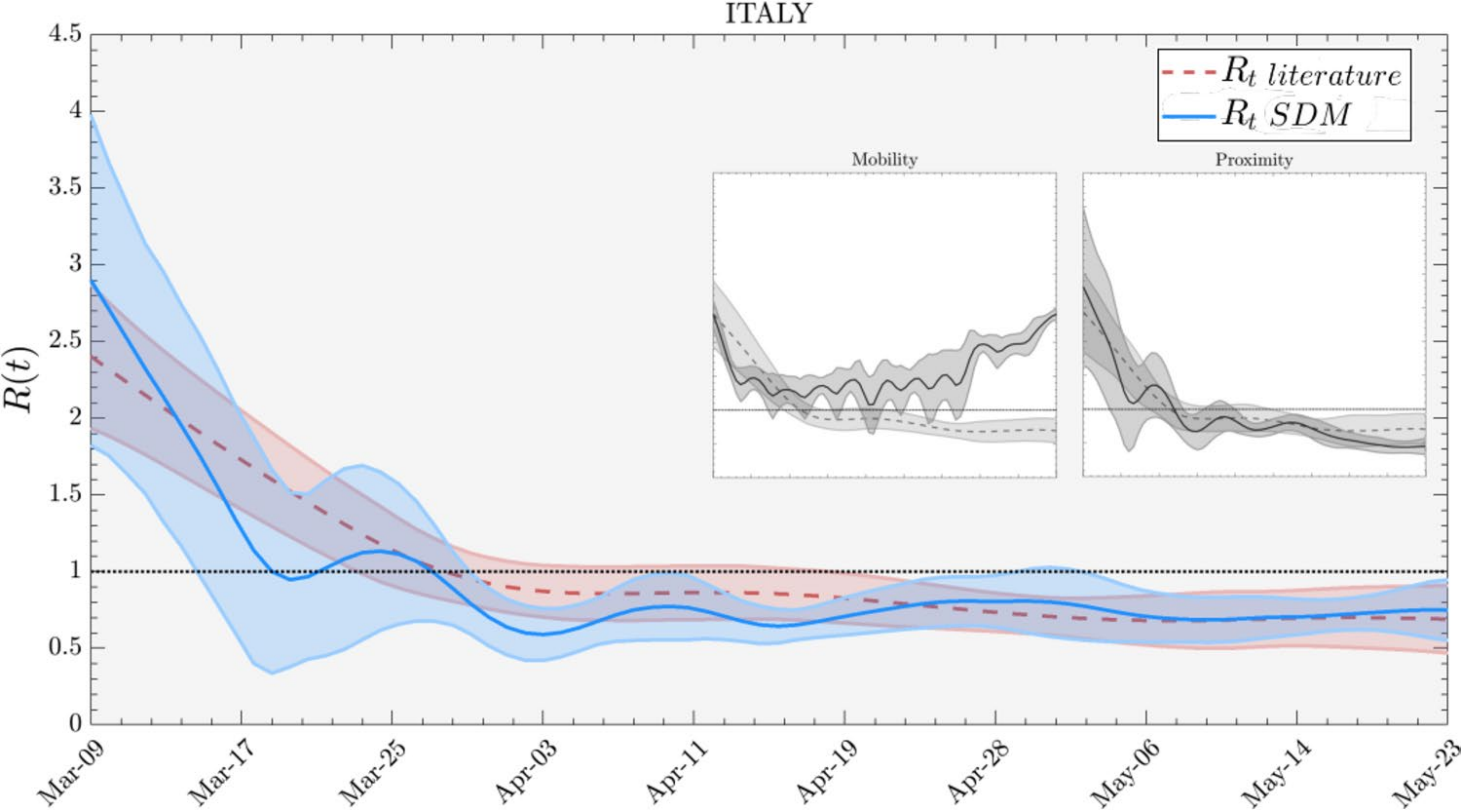
Effective reproduction number for Italy during the lockdown period (March 9th to May 18th). We compare the $R_t$ (dashed red) estimate by well established approaches with $R_t$ SDM (blue) from the method we propose using human mobility data. Includes depletion of the susceptible population, individual mobility and physical proximity. The left inset compares the $R_t$ (dashed) with the $R_t$ SDM (solid) by using mobility data only and the right inset compares the $R_t$ (dashed) with the $R_t$ SDM (solid) by using physical proximity data only. The scale of the insets are the same as the main plot. Calibration coefficient $c=1.14$ (see “Calibration” subsection of Methods).

In the general analysis of epidemic data we refer to reported infected persons by their dates of diagnosis via laboratory test. However, some countries also report infections by the date of first symptoms reported by patients. In particular, we have used the latter type of data when possible (Italy) and inferred it in the case of the USA and the UK via an analysis of the effective reproduction number assessed by^22,27,28^.

We use epidemiological data at the level of states and mobility data at the level of cities for US locations and at the level of state for EU countries. We have studied and analyzed these regions during the period in which lockdown policies were in action as reported in^29^. Finally, for US states, we use^22^ as estimation of the reproduction number as well as the estimation of susceptible population considered. When analyzing other countries, we use various sources, averaging Epiforecast^27^ and Covid19 projections^28^ so as to have an ensemble calculation of the actual reproduction number *R*(*t*).

In Fig. 1, we show the hardest hit states in the US as of June 2020: New York and Florida. Note the good agreement between the theory of this paper using mobility and proximity and independent estimates of the reproduction number. Note that for New York state an important cause for the reduction in *R*(*t*) is due to the depletion of the susceptible population, while physical distancing has a smaller impact. Meanwhile, in Florida, the behavior of *R*(*t*) is mainly due to physical distancing restrictions taken up at the end of the shelter-at-home policy.

**Figure 3:**
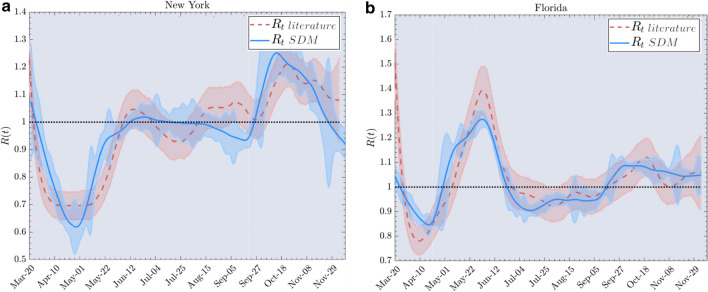
Effective reproduction number for New York (**a**) and Florida (**b**) state in USA for a more extended period of the epidemic, by using data from Google^23^ mobility trend.

The use of appropriate face coverings should reduce the transmission of CoViD-19 by individuals who do not have symptoms and may reinforce physical distancing. Public health officials also caution that face coverings may increase risk if users reduce their use of other efficacious measures such as physical distancing and frequent hand washing. In the singular case of Italy as report in Fig. 2, we take the number of face masks distributed to the population as a proxy for physical proximity (i.e., we assume the number distributed is effectively equivalent to a certain interpersonal distance), since at the beginning of the outbreak Italy has reported the number of distributed face mask in the country^30^.

In Fig. 3, we show the two derivations of the effective reproduction number *R*(*t*). The first is found using RtLive^22^. We use this to study the diffusion of the second wave of CoViD-19 in USA. We have sourced mobility and proximity data from the Data for Good program^31^ (see also the SI section B for further discussions and results). The analysis covers the period from March to November 2020 in two US states (New York and Florida). Our analysis closely matches the epidemic risk trend by using mobility data and new cases yielding an *R*(*t*) value 6 days sooner than typical *R*(*t*) estimations in literature.

**Figure 4:**
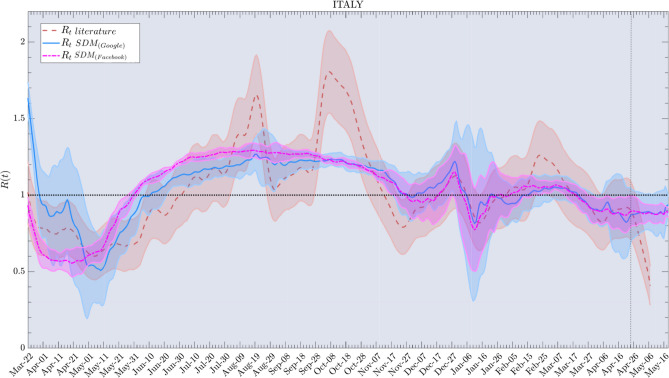
National Effective reproduction number for Italy during the period from March 2020 to May 2021. Here, $R_t literature$ is calculated using the method of^32^. After the vertical dash line this estimate is not based on completed data. Calibration coefficients are $c=1.15$ for the $R_t SDM$ estimate based on Google data and $c=1.17$ for the $R_t SDM$ estimate based on Facebook data, and $\tau _g=12$ (see “Calibration” subsection of Methods).

Alternatively we perform a further analysis comparing our $R_t$ SDM with the reproduction number computed by a direct renewal equation where we use the number of cases by the onset of symptoms, as in Fig. 4, data from^33^ for Italy. In this figure we have used the renewal estimate of the reproduction number as in^16^ and the social distancing based estimation using two dataset for human mobility trend from Google^23^ and^31^. We notice that the anomalous peak observed in the epidemiological estimate in October is not present in the social estimate. This effect is largely due to an abrupt increase of number of performed tests in that period.

Finally, we call attention to the fact that mobility alone is not sufficient to explain the dynamics of epidemics, as discussed in^34^. We see that physical proximity is crucial in resolving why a relatively stable *R*(*t*) below 1 has persisted, despite an increase in mobility after the end of the lockdown period. On the other hand, one should subtract from the susceptible population the number of asymptomatic or undocumented infected individuals, which are not counted in official reports. We provide an estimate for this number in the “Methods” section.

The effects of vaccination on $R_t$ are difficult to estimate. In Fig. 4 we present a preliminary analysis of these effects. Taking into account vaccination is necessary to accurately estimate the SDM reproduction number once a significant fraction of the population has been vaccinated. We assume that vaccination reduces the fraction of the population that is susceptible by a factor of $1-\nu (t)$, where $\nu (t)$ is the fraction of the population that has received the vaccine at time *t*.

It is outside the scope of this paper to explore the effects of vaccination; however, we stress the importance of using this information to properly assess the reproduction number via mobility data. In a follow-up paper, the authors intend to analyze the effects of different types of vaccines on $R_t$ at the regional (and higher) level.

## Discussion

The outbreak of the CoViD-19 pandemic has pushed many countries towards a response that relies on the policy of social distancing, the implementation of which has important social and economic impacts on the organization of production and on the work process. In response to the CoViD-19 pandemic, countries have introduced various levels of ‘lockdown’ to reduce the number of new infections.

From Eq. (2) it is evident that as the epidemic evolves the force of infection is reduced for various reasons, primarily due to physical distancing policies adopted by most countries in the form of a lockdown of human mobility. Since it is not practical to reduce physical distancing beyond a certain socially and economically acceptable level, the only foreseeable reasons for the end of an epidemic are the depletion of susceptible population (immunization), a change in the intrinsic infectiousness of the virus, a sustained change in public hygiene habits (mask wearing, physical distancing, etc.), or innovation in contact tracing, testing, and isolation, see^35^ for a discussion.

Mechanistic models of disease transmission are often used to forecast disease trajectories and likely disease burden, but are hampered by substantial uncertainty in disease epidemiology in the presence of significant social feedback. Models of disease transmission dynamics are hindered by uncertainty in the role of asymptomatic transmission, the length of the incubation period, the generation interval, and the contribution of different modes of transmission.

Infectiousness depends on the frequency of contacts and on the level of infection within each individual. In airborne infections, the former can be decomposed as a product of mobility and physical proximity, interpreted broadly as an effective distance measure which also includes the amount and type of physical protection used. The latter involves an internal micro-scale competition between the virus and the immune system which depends on environmental factors like pollution levels and repeated viral exposure, which can modify the viral load shed by infectious individuals.

We have mainly focused our study on the spread of a contagion in a homogeneous population, using a renewal collisional equation which has proven to be a powerful tool for analyzing and modeling epidemic data along side other well established measurements of the reproduction number. We have found it to be both practically and conceptually useful. This analysis has focused on the lockdown, but the same theoretical tools along with additional technology and data resources show promise for the analysis of the post-lockdown response and further mitigation of this disease.

At this stage, we do not investigate the dynamics of the severity of the disease. In order to examine these dynamics, we would need to focus our attention on the microscale corresponding to viral particles and immune cells. Since these agents induce the dynamics of the varying intensities of the disease observed at the macroscopic scale of the human population.

Furthermore, to assess the severity of an epidemic in a population, one should take into account both the reproduction number *R*(*t*) and the absolute number of cases. A high *R*(*t*) is manageable in the very short run as long as there are not many people sick to begin with. An important aspect of *R*(*t*) is that it represents only an average across a region. This average can miss regional clusters of infection. Another subtlety not captured by *R*(*t*) is that many people never infect others, but a few ’superspreaders’ pass on the disease many more times than average, perhaps because they mingle in crowded, indoor events where the virus spreads more easily. This means that bans on certain crowded indoor activities could have more benefit than blanket restrictions introduced whenever the *R*(*t*) value hits one. In conclusion, in addition to *R*(*t*) one should look at trends in numbers of new infections, deaths, hospital admissions, and cohort surveys to see how many people in a population currently have the disease, or have already had it.

Fatality rates and intensive care hospitalization rates are related to disease severity. In our collisional kinetic framework we have considered contacts among individuals to be random. In addition to these erratic contacts , one can consider structured contacts occurring at home, in hospitals, workplaces, and schools, just to mention a few of the possibilities. For structured contacts, we should consider the use of a different approach than collision theory. One example of a situation in which interactions are more structured is in the theory of random growth of surfaces. In the model considered by^36^, the growing surface is represented as a set of columns, which can be thought of as the individuals of a society that interact. These individuals influence each other and self-organize in the presence of noise so that anomalous scaling and long-range correlations are produced, which are a manifestation of the cooperation among individuals. Since people interact in correlated ways^37^, an extension of the collisional model of the present paper to include correlations among the movements of individuals would be more realistic (and likely important for small population sizes or parameter values near $R=1$ or $R=0$). For a simulation of the interplay between the social and epidemiological effects in a two-layer network, see^38^.

We stress some advantages of using $R_t$ SDM alongside the well-established estimations in literature. First, the social estimate is available a couple of weeks earlier then the epidemiological estimate. Second, if a deviation is observed between these two estimates, it may be a sign of a change in the transmissibility of the virus. Furthermore, the approach we have presented allow us to disentangle the effects of population mobility, physical proximity, and depletion of susceptibles on the progression of the epidemic. Knowing the effects of each of these components of the response of the government and society to the CoViD-19 epidemic should allow for less costly and more effective strategies for defeating and mitigating epidemics. In particular, this collision model approach to estimation of infection spread should help policy makers and governments to better assess the continuing threat of CoViD-19 to the public welfare.

## Methods

The renewal equation was introduced in the context of population dynamics studies. Later it was reinterpreted along the lines of stochastic processes, as in^39^, where transmission occurred via a Poisson infection process. This process is such that the probability that, between time *t* and $t+\delta t$, someone infected a time $\tau$ ago successfully infects someone else is $A(t, \tau )\delta t$ , where $\delta t$ is a very small time interval. As a consequence, the predicted mean infectious incidence at time *t* follows the so-called renewal equation:

3 $$\begin{aligned} j(t)=-\frac{d}{dt}n_s(t)=\int _{0}^{\infty }A(t,\tau )j(t-\tau )d\tau +i(t) \end{aligned}$$

where $\tau$ is known as the infection age and *j*(*t*) is the rate of production of infectious individuals. The kernel $A(t,\tau )$ is the average rate at which an individual infected $\tau$ time units earlier generates secondary cases. In other words, $A(t,\tau )$ is the expected infectivity of an individual with infection-age $\tau$, it can be interpreted as the reproduction function for new infections at time *t*. A practical issue concerns the extrinsic dynamics (e.g., public health interventions) of time inhomogeneities in the number of cases highlighting the depletion of susceptible individuals when contact tracing, quarantine, and isolation are implemented during the course of an epidemic. Finally, *i*(*t*) is a function that describes the effects of an external source of infected persons. For the special case $i(t) = A\delta (t)$, it encodes the initial number of imported infected individuals. Let us notice that one could completely disregard the external source of infectious individuals, by modelling an infinitely old epidemic where $\tau \in [0,\infty )$ in the renewal integral so disregarding the imported cases.

The kernel *A* can be factorized as

$$\begin{aligned} A(t,\tau )=n_s(t)\beta (t,\tau )\Gamma (t,\tau ), \end{aligned}$$

where $\beta (t,\tau )$ is the product of the contact rate and the risk of infection (i.e., the effective contact rate), and $\Gamma (t,\tau )$ is the probability of being infectious at infection age $\tau$. So, reduction in contact frequency with calendar time *t* affects $\beta (t, \tau )$ while early removal of infectious individuals at calendar time *t* changes the form of $\Gamma (t, \tau )$. An earlier average infection age at first transmission of the disease will result from contact tracing and isolation. However, the classic approach to renewal equations for epidemics assumes, as in^12,40–42^, that the non-linearity of an epidemic is characterized by the depletion of susceptible individuals alone, so that

$$\begin{aligned} A(t,\tau )=n_s(t)\beta (\tau )\Gamma (\tau ). \end{aligned}$$

Finally, the proportion of persons who have the ability to infect at a given calendar time is given by the number of infected individuals which is called prevalence,

$$\begin{aligned} p(t)=\int _{0}^{\infty } \Gamma (\tau ) j(t-\tau ) d\tau . \end{aligned}$$

Notice that *p*(*t*) is not the number of active infected individuals generally reported in epidemic data published by different national health services. This is because the officially detected cases are actively confined (in hospitals or at home) and so their contribution to the spread of the epidemic is not so relevant. On the contrary *p*(*t*) represents the infected people that are still conducting their lives as usual, possibly infecting other people.

The most important assumptions in our use of phenomenological models are (1) Short time scale of the epidemic (much shorter than the characteristic birth and death time scales of the population) (2) Well mixed population (force of infection homogeneously the same for all ages, sexes, etc.) (3) closed population (no immigration or emigration) (4) initial small shock (the initial infected group extremely small with respect to the size of the susceptible population).

Using the collision theory for chemical reactions in solution with two types of molecules, we can write down the rate of contacts between the two types in a given volume, per unit time $z= n_s j_{\text {x}} 2\pi r \nu$. Where we have assumed that all agents are ideal point particles that do not interact directly, and travel through space in straight lines. We further investigate the assumption of such collision model in^43^. However, not all contacts will result in secondary infectious, rather only those contacts that have sufficient viral load so as to surmount a certain threshold for triggering the infection. Such transmission efficacy should depend inversely on the physical distance between individuals. Moreover, the collision rate, in reality, depends on time and, in general, on the epidemic’s evolution. This is because the total number of agents changes over time. As an approximation, we embed all of these complexities in the choice of the radius *r*, so to maintain the simplest form of crossection *z*.

Suppose that during an outbreak only a certain fraction of infectious persons are observed through direct testing, other infectious individuals are not observed, e.g., because of lack of symptoms or the mildness of their illness. In particular, asymptomatic secondary transmissions, caused by those who have been infected and have not developed symptoms yet, and also by those who have been infected and will not become symptomatic throughout the course of infection, must be considered. At a given calendar time, *t*, we imagine that the important new cases are not the observed newly infected (which are quarantined or self-isolate), but rather the fraction of newly infected that are not observed. Some of these unobserved infected spread the disease. The observed cases are a fraction $\lambda _t$ of all cases, i.e.,

$$\begin{aligned} j_o(t)=\lambda _t \,j(t), \end{aligned}$$

where $\lambda _t$ is the rate of detection which can change over time depending on the details of and degree of adherence to testing protocols and medical screenings. Moreover, the observed cases together with the undocumented cases constitute all cases so that

$$\begin{aligned} j(t)=j_o(t)+j_{\text {x}}(t). \end{aligned}$$

Thus, the relation between undocumented infected and documented infected individuals is

$$\begin{aligned} j_{\text {x}}(t)= j_o(t)\frac{1-\lambda _t}{\lambda _t}. \end{aligned}$$

If the population screening procedure is effective, we have $\lambda =1$. This could happen, for example, if the infected group is made up of only symptomatic persons which are infectious only after the onset of symptoms. As a first approximation, we have considered $\eta$ to be constant over the time periods we considered, and $\lambda$ to be a slowly changing function (over a time scale of $\tau _A$ with respect to the calendar time *t*) so that $\lambda (t-\tau )\approx \lambda (t)$.

Finally, the actual (or effective) reproduction number can be written as the incidence-prevalence ratio

$$\begin{aligned} R(t)= D \frac{j(t)}{p(t)}, \end{aligned}$$

where prevalence is the proportion of persons who have the ability to infect at a given calendar time. This ratio indicates the propensity of currently infected individuals to infect susceptibles and

$$\begin{aligned} D:=\int _{0}^{\infty }\Gamma (\tau )d\tau \end{aligned}$$

is the average infectious period (or mean generation time). Therefore the actual reproduction number written as incidence persistence ratio is:

4 $$\begin{aligned} {R(t+\tau _g)}\sim R(t_0+\tau _g) \dfrac{n_s(t)}{n_s(t_0)}\dfrac{\eta (t)}{\eta (t_0)}\dfrac{\rho (t_0)}{\rho (t)} \dfrac{\nu (t)}{\nu (t_0)} \dfrac{ 1-\lambda (t)}{ 1-\lambda (t_0)} \equiv {R}_0 {\tilde{S}}_t\tilde{\mathscr {B}}_t, \end{aligned}$$

where we have also considered some practical issues in the calculation of the reproduction number as given in^18^.

Note that, *R*(*t*) does not depend explicitly on $\Gamma (\tau )$, except through its integral over all possible values of $\tau$. Thus, to a an adequate degree of approximation, it only depends on the typical time between infection and detection. Indeed, one can replace the $\Gamma$ distribution with any distribution with the same mean recovery time (i.e., time to become non-infectious). As a consequence, the most changing $\Gamma$ (as a function of $\tau$) can do is change *R*(*t*) by a re-scaling. However, effectively, the infectious age distribution depends on *t*. Since contact tracing, testing, and isolation (as well as treatments) will tend to reduce the active infectious period (and their use depends on *t*). On the other hand, the absolute scale of *R*(*t*) is also important, since one would like to maintain a value of *R* below 1.

### Calibration

We discuss some important points for the calibration of the social distancing estimate of $R_t$. First, calibration is required since we need to align the SDM reproduction number we compute with the reproduction number derived using the epidemiological data obtained by estimation methods in literature. Regressing the two variables, we find a constant *c* which is then embedded in the the ${\hat{R}}_0$. The second point is essentially due to possible misalignment between the two different estimation procedures because of intrinsic discrepancies in data we use. For this purpose, we evaluate the multiplicative scaling factor in the reproduction number Eq. (4), using a zero-intercept linear regression between the two time series of the reproduction number.

Additionally, we have set the generative time $\tau _g=6$, which takes into account the typical time to generate positive test results. This time scale is interpreted as detection period which is the time between exposure (contact) and detection (isolation)^44–46^. The generative time has been estimated through the synchronization between the signals in the calibration steps. This is equivalent to the delay estimate used by RtLive and Epiforecast and other reconstruction infection estimates as^17,18,47,48^ as in Fig. 3. Note that for Fig. 4 we used the method of Ref.^32^ to estimate *R*(*t*). Since this estimate was not adjusted for the delay in reporting symptoms, it was necessary to add an additional 6 days to the value of $\tau _g$ for a value of $\tau _g=12$. The values chosen produce a good alignment between the features of *R*(*t*) derived as in the literature and corresponding features of *R*(*t*) as given by Eq. (4).

A last step in the calibration consists in the estimation of the fraction of the population that is infected, which is particularly important for a longer-term analysis has. This is accomplished by studying the dependence of the reproductive number *R*(*t*) on the ratio, *c*(*t*), between the official number of people infected and the total population of the region (Italy) or state (US)^16^. The value $\lambda$ has changed over time throughout the epidemic and after the end of the spring 2020 lockdowns it increased, possibly due to the increased number of tests performed. Finally, when plotting the reproduction number, to visualize the trend, we use non-parametric regression analysis with LoWeSS (Locally Weighted Scatterplot Smoothing) surrounded by a $90\%$ confidence interval obtained through bootstrapping.

## Supplementary information

Supplementary Information 1.
