# Supplementary material for: On the use of aggregated human mobility data to estimate the reproduction number
Source: Sci Rep. 2021 Dec 2;11:23286. doi: 10.1038/s41598-021-02760-8 (PMC8639785; doi:10.1038/s41598-021-02760-8)
Supplement: Supplementary file 1 — Supplementary Information 1. [file 41598_2021_2760_MOESM1_ESM.pdf]

On the use of aggregated human mobility data to estimate the reproduction number

Fabio Vanni, David Lambert, Luigi Palatella and Paolo Grigolini

November 13, 2021

A Data description and estimation analysis

The data repositories used to obtain our results are listed in Table 1. This table includes both the epidemiological and social distancing data sources. We follow two approaches in evaluating proxies for physical proximity. The first is in regard to the Italian evolution of the CoViD-19 epidemic along the lines of social distancing trends. The rest of the studies address the course of the epidemic in selected US states and three European countries: the UK, Ireland, and the Czech Republic, for which data on physical proximity were available to us.

Table 1: Repositories used for epidemiological and social distancing data. Estimates for the reproduction number are reported along the paper as estimations in literature.

**Data Repositories**
Epidemiological
Johns Hopkins University [1]
CoViD Tracking Project [2]
Dip. Protezione Civile [3]
Epicentro ISS [4]
Gov.UK CoViD-19 [5, 6]
Social Distancing
Google Mobility [7] (*mobility* and *proximity*)
Voxel51 Physical Distancing Index [8] (*proximity*)
Unacast Social Distancing Dataset [9] (*mobility and proximity*)
The COVID-19 Mobility Data Network [10] (*mobility and proximity*)
Analisi Distribuzione Aiuti [11] (*Personal Protective Equipment*)
$R(t)$ estimations
Rt live [12] (USA only)
Epiforecast [13]
CoViD-19 Projections [14]

Recall the social estimate of the reproduction number:

$$R(t) \sim R(t_0)\frac{n_s(t-\tau_g)}{n_s(t_0-\tau_g)}\frac{\eta(t-\tau_g)}{\eta(t_0-\tau_g)}\frac{\rho(t_0-\tau_g)}{\rho(t-\tau_g)}\frac{\nu(t-\tau_g)}{\nu(t_0-\tau_g)}\frac{1-\lambda(t-\tau_g)}{1-\lambda(t_0-\tau_g)}. \tag{1}$$

There are many definitions of mobility and a great variety of data repositories. Typically, mobility trends are subdivided into movement trends and trends in distance traveled. The first mobility proxy accounts for changes in movement fluxes for which we have relied on Google mobility open source data [7]. Google reports movement trends as reflected in changes in numbers of visits to various locations (parks, public transportation hubs, residences, etc.), relative to an average baseline on the same day of the week evaluated before the pandemic outbreak. We have used the the average mobility across all locations (residential included).

The second type of mobility proxy is the percent change in average distance traveled, as Unacast [9]. When kilometers traveled per day is used as a mobility proxy, one can use the principle of maximum entropy, under the assumption that the individuals are independent particles and that there is an average daily trip length in the population. Thus, the distribution of distances travelled is exponential,

$$p(L) = L_0 e^{-\frac{L}{L_0}}.$$

Here L_0 is the characteristic daily path length reported in mobility data. We checked that distance traveled per day and movement requests show almost exactly the same trend. These data offer metrics for physical distancing based on GPS devices. They provide proxies for social mobility (interpreted as average distance traveled) and interpersonal proximity (human encounters), calculated with respect to the 4 weeks before the CoViD-19 outbreak. The former is the percent reduction in the total distance traveled per device, averaged across all devices located in a given US state. The latter is an estimate of close encounters between two devices per square kilometer, expressed as a fraction of the baseline. This estimate counts two users from the same province as having come in contact if they were within a circle of radius 50 meters of each other within a 1-hour period.

Regarding physical proximity we use various proxies according to the availability of data in the aforementioned repositories, namely the Physical Distancing Index (PDI) of Voxel51 [8] and the Human Encounters Index [9]. Voxel51's estimates depend on deep learning models that are able to detect and identify pedestrians, vehicles, and other human-centric objects in the frames of each live street cam video stream in real-time. Using images sampled from each video stream every 15 minutes, they compute the PDI, an aggregate statistical measure that captures the average density of human activity within view of the camera over time. The PDI value for a particular day will not exactly correspond to the number of people in that exact frame. Rather, the PDI value at time, t, is an average measure of peak activity during a window of time (a few days) around that time. So, a large PDI value means that there were a lot of people out and about around that time, at some point.

Voxel51's PDI website presents measures of activity at a single location in the city. While these measures are likely correlated with overall trends in activity in the city, and are thus an interesting proxy for public behavior, this is not guaranteed. This data on the concentration of people in a certain area lets us estimate the density of individuals in a two-dimensional plane. From that information, one can infer the mean distance between individuals, assuming random positions. Note that this proxy has been obtained at the level of cities.

Another proxy for physical proximity is provided by the Unacast dataset [9]. These data offer metrics for physical distancing based on GPS devices. They provide proxies for social mobility (interpreted as average distance traveled) and interpersonal proximity (human encounters), calculated with respect to the 4 weeks before the CoViD-19 outbreak. The former is the percent reduction in the total distance traveled per device, averaged across all devices located in a given US state. The latter is an estimate of close encounters between two devices per square kilometer, expressed as a fraction of the baseline. This estimate counts two users from the same province as having come in contact if they were within a circle of radius 50 meters of each other within a 1-hour period.

In order to turn the estimate of the number of close encounters into an estimate of proximity, we use the principle of maximum entropy. The maximum entropy distribution of particle positions would be a uniform distribution. This corresponds to an exponential distribution of inter-particle distances. The characteristic length scale (equal to the mean distance between particles) is the inverse of the square root of the particle number density (up to a factor of order π). A similar derivation was given by [15], where the proximity ρ to the first nearest neighbour in a randomly distributed population at density δ, can be written as

$$\rho = \frac{1}{\sqrt{\delta}}. \tag{2}$$

At this point we show an example of how to use Facebook mobility data on the estimation of the social reproduction number R_t. Facebook provides two types of indicators: the percentage change in movement relative to baseline and proportion of users staying put within a single location. The first variable is used as a proxy of movement change transforming it to a decimal number with baseline of value 1 instead of 0. As for the second variable, we take the complement to 1 in order to get an estimation of how many people are not at home. After such normalization, in order to obtain a proxy for proximity, we transform the normalized second variable (which is δ) according to Eq. (2). Then we plug those variables directly into Eq. (1). A very similar approach is taken for the other types of data from Google, Unacast and Voxel51.

Moreover, for the case of Italy, we also use data on the number of face coverings and masks distributed among the population, which we interpret as inversely proportional to physical proximity in the model. This should be reasonable, at least during the period of lockdown. MacIntyre [16]and Chu [17] show that the risk of getting infected drops by half for every additional meter of distancing.

In Figs. 1-2, we plot the reproduction number, making use of Google [7] mobility and Voxel51 [8] proximity data for certain US states and EU locations.

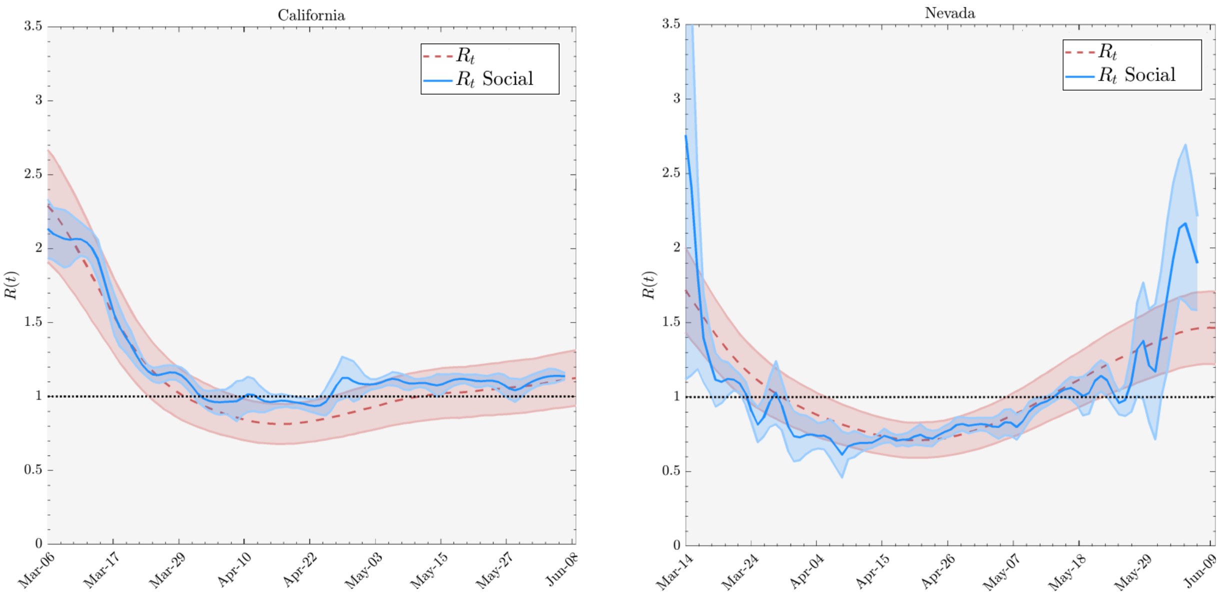

(a) (b)

Figure 1: Reproduction number estimates for two US states: California (evaluated in Los Angeles) (a) and Nevada (evaluated in Las Vegas) (b). In the latter case the camera was in a location devoted to leisure activities, this yields a biased estimate of the mean interpersonal proximity as compared with rest of the state.

A selection of results of $R(t)$ via social distancing is shown in Figs.3-7 for many US states, using Unacast [9] mobility and proximity data for the first period of the epidemic from March to July 2020. These results are consistent with

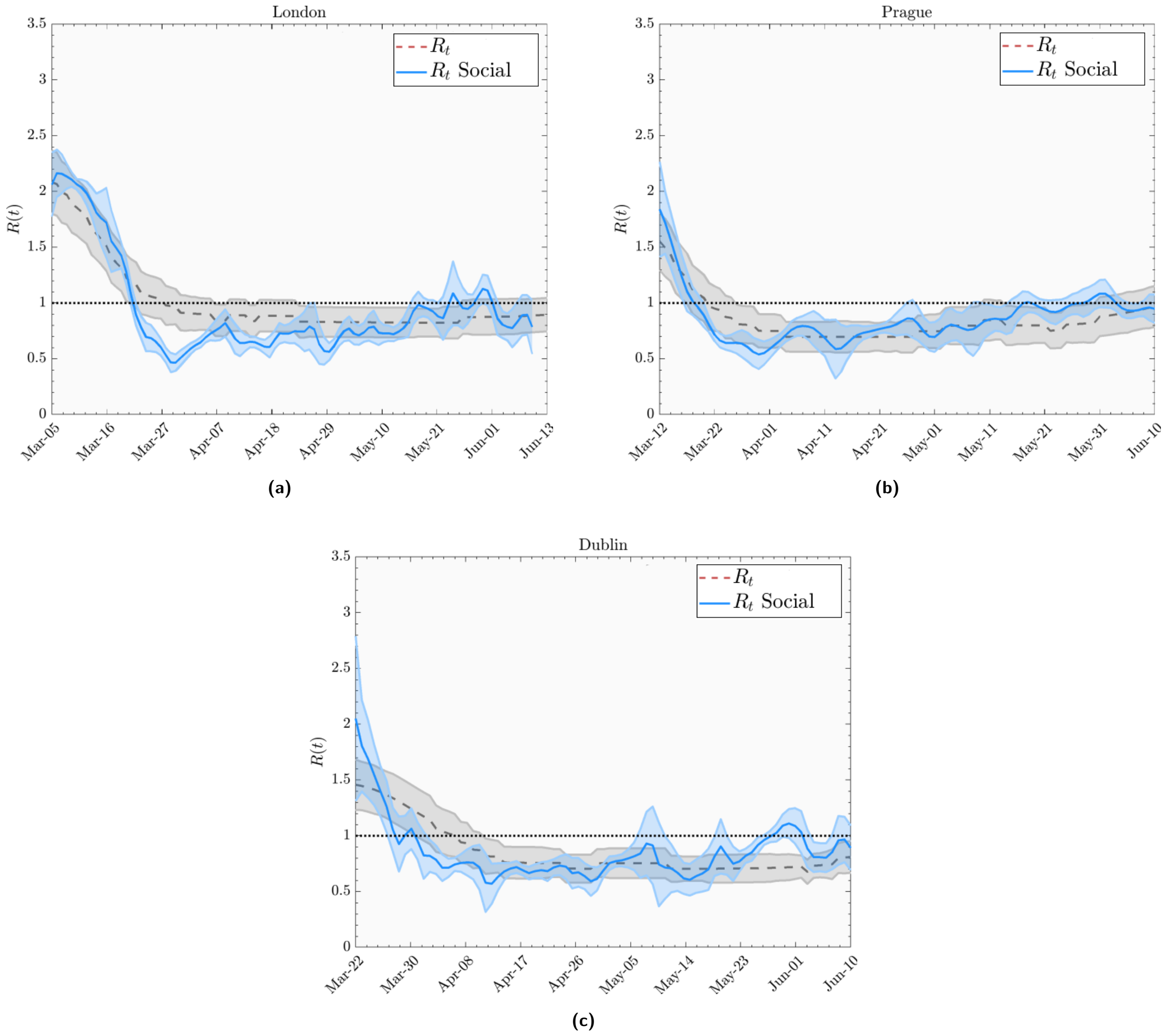

Figure 2: Reproduction number estimates for London, UK (a), Prague, Czech Republic (b), and Dublin, Ireland (c). Comparison between the reproduction number ensemble average of [13] and [14] (gray dashed line) and the reproduction number computed according our kinetic approach, using data from [7] mobility, [8] for social proximity and [2] for epidemic data. Ribbons are the 80% credible interval obtained via bootstrap. Epidemiological data and $R(t)$ estimation are from data referred to the date of lab diagnosis.

the those obtained using other databases for social mobility and human proximity.

In order to confirm our analysis also for the second wave of CoViD-19, we apply the approach presented above for the second wave of epidemic in Italy, for the period from September 2020 through December 2020. In this period we have used data from Facebook's database, and infer both mobility and proximity from movement range data as described in [10]. We have considered level changes in the rates of mobility and staying put. The first measures the change in frequency of travel between the last two weeks of February and the present day. The latter indicator measures the percentage of people who remained in one location for 24 hours with respect to the same baseline period. One minus this value is a proxy of the density of people exhibiting movement outside the vicinity of their home, and that can propagate the contagion. We have made the same estimate with Voxel51 and Unacast databases, which we have used for our analysis through the period of June/July 2020. These two data providers exited the Data for Good program after summer 2020, so for the second wave analysis, we have preferred to use another data provider which still participated in the Data for Good program. The CoViD-19 Mobility Data Network has helped us confirm the results we found for the first part of the pandemic. We set the beginning of the second wave in

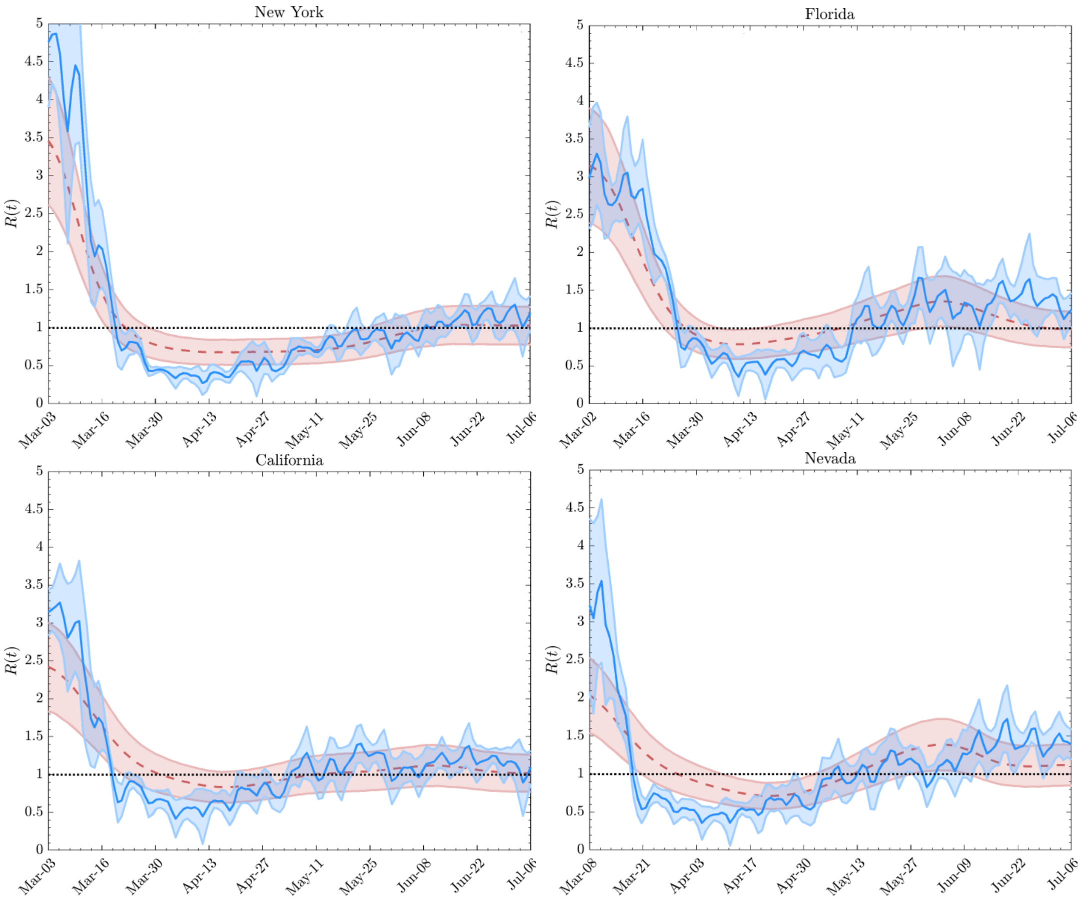

Figure 3: Reproduction number of CoViD-19 as estimated in literature, red, and via social proxies (see Eq. 1), blue, for New York, Florida, California, and Nevada. Effective reproduction number data courtesy of Rtlive [12] and social distancing data courtesy of Unacast [9].

Europe at September 22nd 2020 following Epiforecast models [13]. However, especially for the CoViD-19 second wave, other important considerations need to be taken into account. For example, virus mutations can trick the ability to efficiently test people and prevent detection of infectious individuals. Furthermore, imported cases need to be taken into account during the periods in which European countries reopened internal and international borders. In summer 2020, the number of imported cases was greater than that of internal cases, so that hubs of infection became crucially important. Consequently, the assumptions of our model cannot entirely hold true. However, after the transient interval of the second outbreak, these clustering effects stabilized. In the specific case of Italy, social distancing interventions have been assessed at the regional level. Notice that the estimation of real cases for the second period is $\lambda \approx 0.12$ for Italy. In the USA, we observe a more continuous trend of R_t without a clear distinction between different waves. So the effects of imported cases and infection hubs play a minor role. In fact, we rely on the Rtlive [12] database provided continuously since March 2020, as reference for epidemiological reproduction number estimations. Thus, we can apply Eq. (1) using mobility and proximity from Facebook's [10] data program. However, we needed to estimate the test and trace variable λ multiple times over the entire period from the end of March 2020 to the beginning of December 2020. In addition to these evaluations, we also apply model calibration for each of the λ intervals. We have applied the overall estimation to some of the hardest hit states in Fig. 8, were we have used $\lambda_1 \approx 0.04$ for the period March-May, $\lambda_2 \approx 0.11$ for the period June-September and $\lambda_3 \approx 0.36$ for the period October-December. In addition, this overall analysis requires a more precise alignment between social distancing and epidemiological data for such an extended period of analysis and calibration via regression multiple times each period.

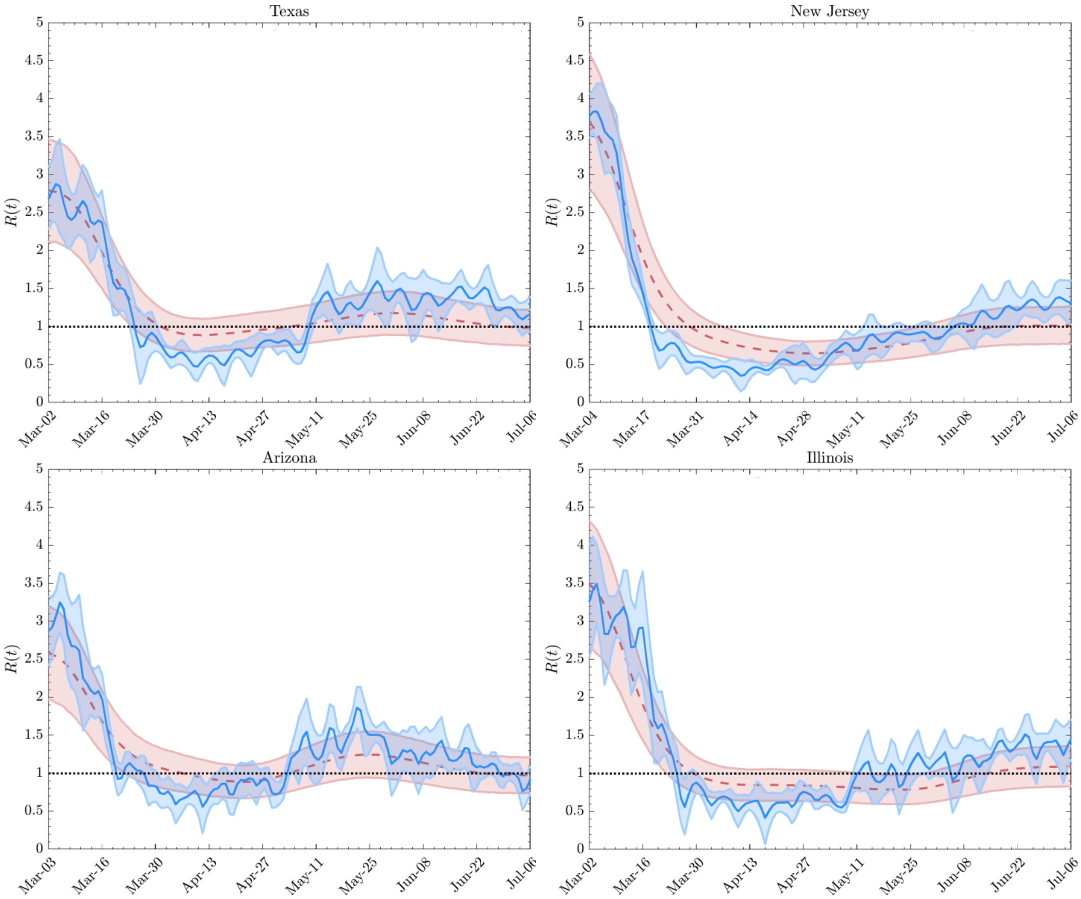

Figure 4: Reproduction number of CoViD-19 as estimated in literature, red, and via social proxies (see Eq. 1), blue, for Texas, New Jersey, Arizona, and Illinois. Effective reproduction number data courtesy of Rtlive [12] and social distancing data courtesy of Unacast [9].

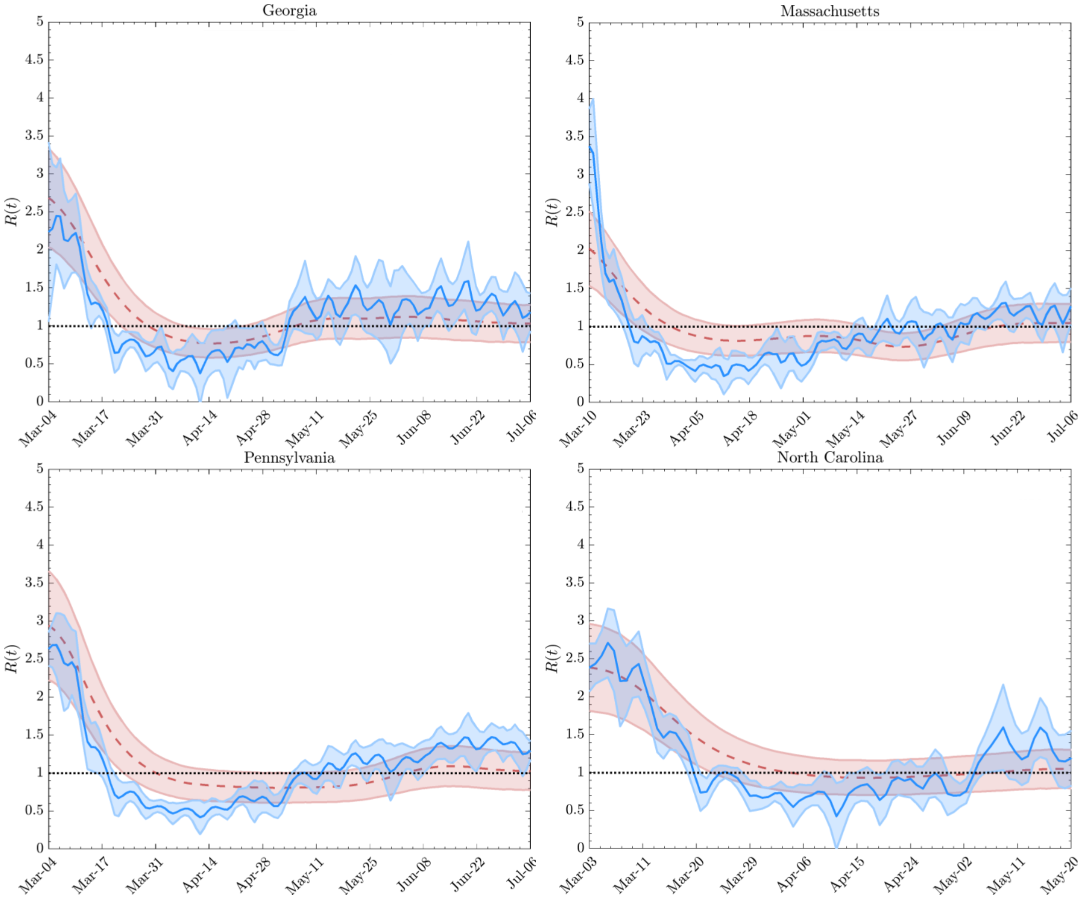

Figure 5: Reproduction number of CoViD-19 as estimated in literature, red, and via social proxies (see Eq. 1), blue, for Georgia, Massachusetts, Pennsylvania, and North Carolina. Effective reproduction number data courtesy of Rtlive [12] and social distancing data courtesy of Unacast [9].

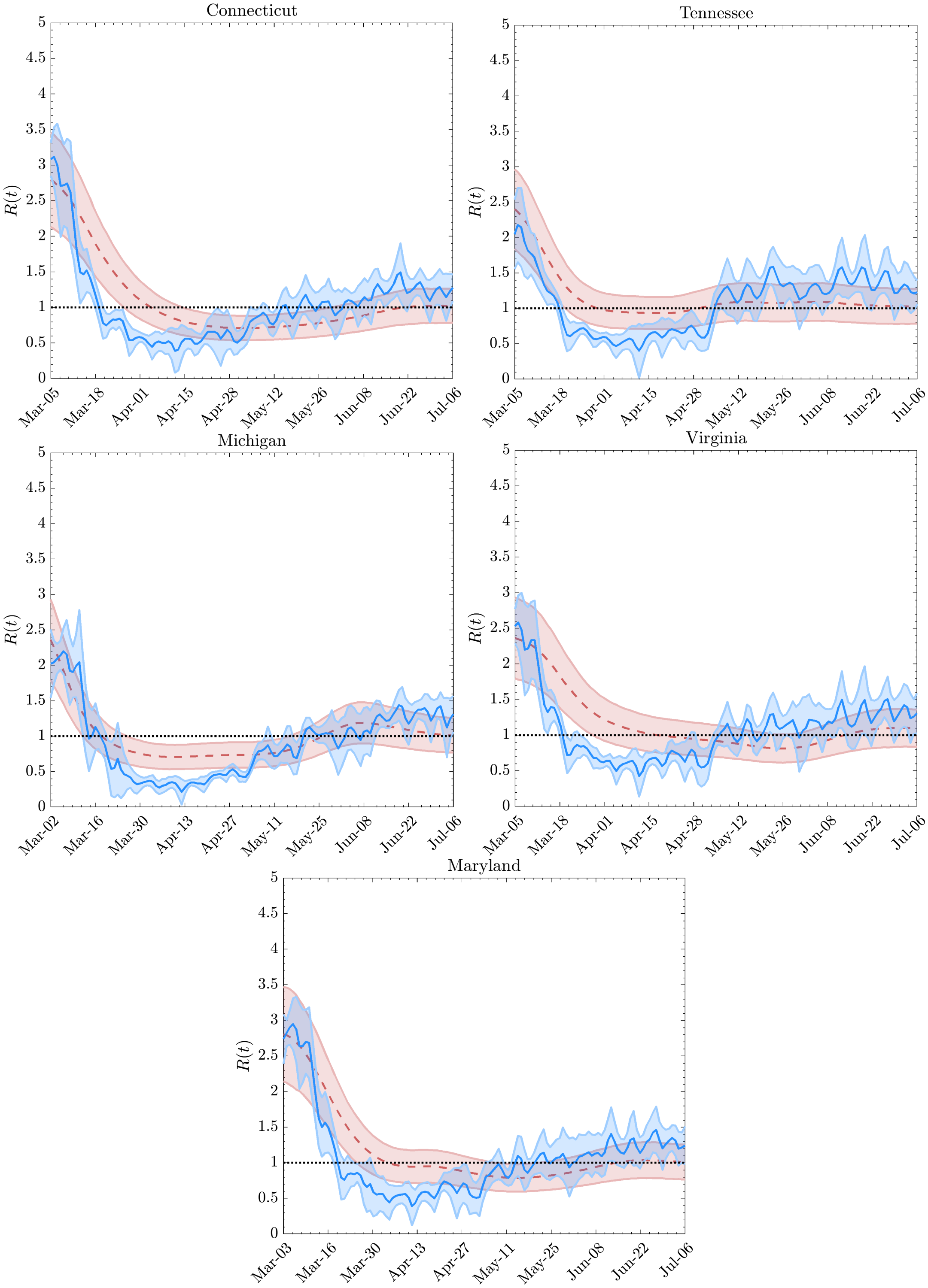

Figure 6: Reproduction number of CoViD-19 as estimated in literature, red, and via social proxies (see Eq. 1), blue, for Connecticut, Tennessee, Michigan, Virginia, and Maryland. Effective reproduction number data courtesy of Rtlive [12] and social distancing data courtesy of Unacast [9].

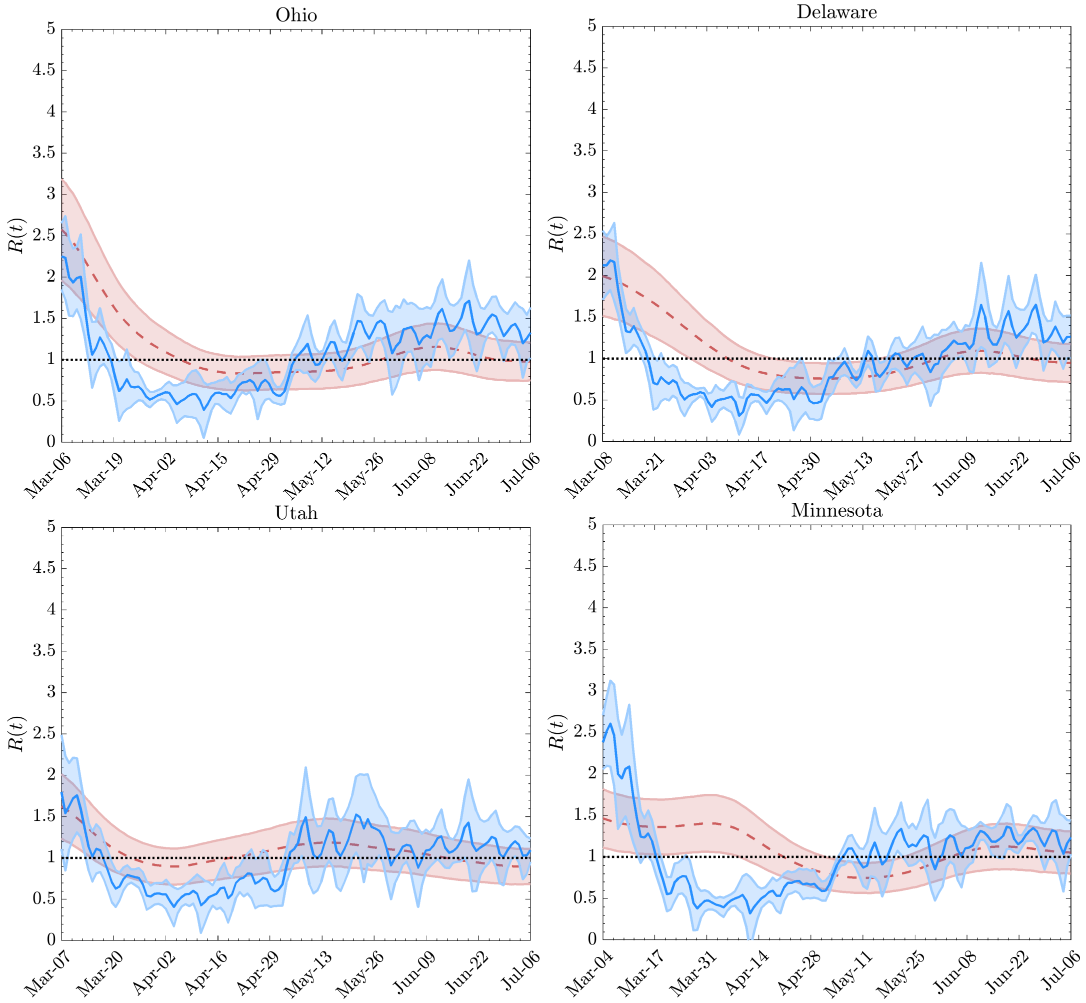

Figure 7: Reproduction number of CoViD-19 as estimated in literature, red, and via social proxies (see Eq. 1), blue, for Ohio, Delaware, Utah, and Minnesota. Effective reproduction number data courtesy of Rtlive [12] and social distancing data courtesy of Unacast [9].

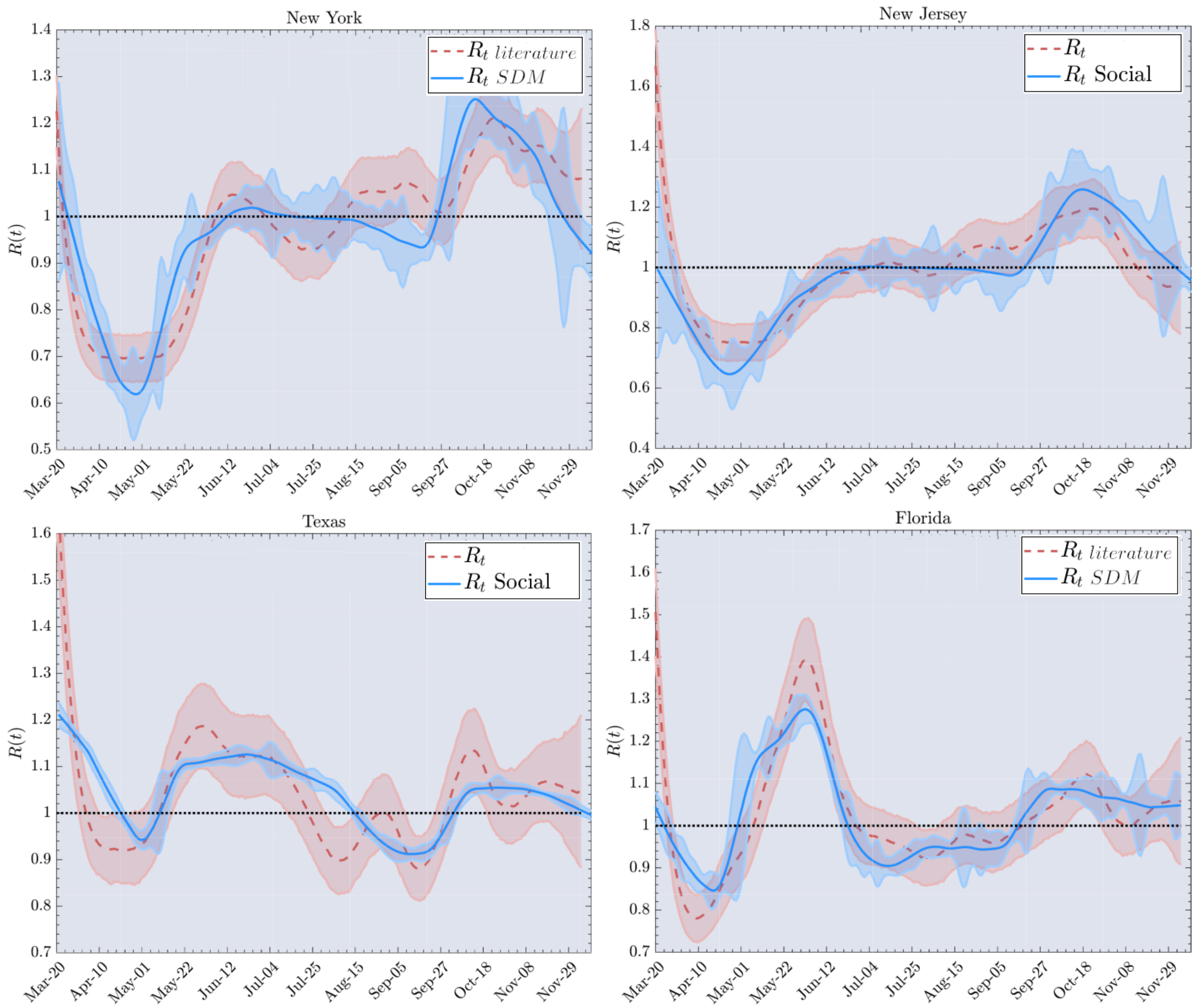

Figure 8: Reproduction number of CoViD-19 as estimated in literature, red, and via social proxies (see Eq. 1), blue, for New York, New Jersey, Texas, and Florida. Effective reproduction number data courtesy of Rtlive [12] and social distancing data courtesy of Facebook [10].

B Description and interpretation of the model variables

Human mobility and interaction radius

We observed above that we used various social distancing data sources as proxies of human mobility and social proximity in our model. In general, one can use any data source for human mobility ν that informs one of the movement trends of the population of a region. We infer it in three ways: km traveled per day by individuals, change in frequency of travel, and number of visitors per place (mobility fluxes) with respect to a baseline period. In general, these are metrics that measure the amount of movement occurring within a population. We consider such movements to be a proxy for average relative speed among particles of an ideal gas.

As for interpersonal proximity ρ, it has an inverse effect on infectivity to that of the interaction radius r. A large interaction radius corresponds to requiring a small distance between individuals for a possibly contagious contact to occur. Assuming people are positioned randomly in a region, we consider that the distance between individuals to be exponentially distributed. Therefore, they have a characteristic interpersonal proximity which is inversely proportional to the number of active individuals in the region of collisions. In this framework, we have inferred social proximity by the number of active individuals given as the complement of people staying at home, or a direct observation of individuals in a certain area.

Test and Trace variable

Changing trends in the reproduction number may be due to several interrelated reasons apart from physical distancing policies. These reasons can be collected into two groups. The first has to do with the virus itself and its capacity to spread. For example, an increase in temperature or the development of less dangerous strains can decrease the effective infectiousness of the contagion. Another hypothesis is that a non-negligible fraction of the population has some form of cross-immunity against SARS-CoV-2 [18, 19, 20]. This could explain why serological tests performed in Italy and USA gave a value of about $\lambda \simeq 0.16$ [21, 22, 23]. The other group of reasons is connected to the decrease in the number of susceptible individuals. Supposing that the latter is the actual reason for changes in R, we fitted it with a linear function of the total population infected using a very simple ansatz:

$$R(t) = \tilde{R}\left(1 - \frac{c(t)}{\lambda}\right). \tag{3}$$

where $\tilde{R}$ is the reproduction number at the beginning of the period of analysis. In this way, when the number of susceptibles approaches zero $R(t) \to 0$. So, the value of the officially detected fraction of the population leading to $R(t) = 0$, $c_{\text{null}} = \lambda$, gives the ratio, λ, between the number of officially detected and the number of actual cases (supposing that this ratio is approximately constant in time). A more detailed and extended discussion of this prevalence estimate from primary data is presented in [24]. These results are *a posteriori* partially confirmed by some preliminary results from antibody testing performed in Italy. They are also in line with the estimated test fraction, λ, found through models used by officials to decide on policies like shelter-in-place orders, such as [25] and [26].

Transmissibility variable

Let us discuss briefly the meaning of the transmissibility variable η defined as the probability that a contact results in an infection. This variable can change with environmental conditions as well as viral infectivity. All viruses,

including SARS-CoV-2, accumulate mutations as they evolve over time. Most mutations have no detectable effect on the biology of the virus. But a few have the potential to change the virus in minor ways and be spread to other hosts. Occasionally, a mutation will enable faster replication, transmissibility, or escape from the immune system, in which case the mutated virus may spread widely throughout the population. However, the D614G variant of SARS-CoV-2 became the dominant form of the virus by March 2020 and continues to be the main form of the virus seen in most infections [27]. Moreover, environmental changes can have an impact on the transmissibility of the virus, as in the case of air temperature, humidity, UV radiation, air flow, etc. All such information is embodied in the variable η which we have taken to be constant over the periods under examination. Theoretically, it would be possible to embed virus-host changes as well as environmental influences in the transmissibility variable η as studied in [28, 29**?**]. In fact, we can link the ability of the virus to be transmitted with a probability of success depending on viral fitness and system "temperature" for crossing immune and environmental barriers, as described by kinetic studies on epidemiologicy as in [28, 30, 31]. However, the estimation of transmissibility η goes beyond the scope of the present work, and we assume it to be stable over the time span of the epidemic. Some variation in η can be accounted for in the calibration process of our analysis.

References

[1] Johns Hopkins University and Medicine. Coronavirus resource center, 2020. https://coronavirus.jhu.edu/.

[2] CovidTracking. The covid tracking project, 2020. https://covidtracking.com/.

[3] DPC. Covid-19 italia, 2020. Sito del Dipartimento della Protezione Civile - Presidenza del Consiglio dei Ministri, https://github.com/pcm-dpc/COVID-19.

[4] ISS. Integrated surveillance of covid-19 in italy, 2020. Scientific coordination by Centro Nazionale per la Prevenzione delle malattie e la Promozione della Salute, CNAPPS - ISS,https://www.epicentro.iss.it/en/coronavirus/.

[5] Gov.UK. Covid-19 in the uk, 2020. available at https://coronavirus.data.gov.uk/.

[6] Greater London Authority. Covid-19 data and analysis, 2020. available at https://data.london.gov.uk/topic/covid-19.

[7] LLC Google. Google covid-19 community mobility reports, 2020. data retrieved from, https://www.google.com/covid19/mobility.

[8] Voxel51. Measuring the social impact of the coronavirus pandemic, 2020. https://pdi.voxel51.com.

[9] Unacast. Unacast social distancing dataset, 5/27/2020. available at https://www.unacast.com/data-for-good.

[10] Facebook. The covid-19 mobility data network, 12/20/2020. available at https://research.fb.com/blog/2020/06/protecting-privacy-in-facebook-mobility-data-during-the-covid-19-response/.

[11] DPC. Analisi distribuzione aiuti (ada), 2020. Sito del Dipartimento della Protezione Civile - Presidenza del Consiglio dei Ministri, http://www.protezionecivile.gov.it/attivita-rischi/rischio-sanitario/emergenze/coronavirus/materiali-distribuiti-alle-regioni.

[12] Kevin Systrom, Thomas Vladek, and Mike Krieger. Project title. https://github.com/rtcovidlive/covid-model, 2020.

[13] Epiforecast. Covid working group, cmmid, 2020. https://epiforecasts.io/covid/.

[14] Covid19Projections. Covid-19 projections using machine learning, 2020. https://covid19-projections.com/.

[15] Stephen P Hubbell, Fangliang He, Richard Condit, Luís Borda-de Água, James Kellner, and Hans Ter Steege. How many tree species are there in the amazon and how many of them will go extinct? *Proceedings of the National Academy of Sciences*, 105(Supplement 1):11498–11504, 2008.

[16] C Raina MacIntyre and Quanyi Wang. Physical distancing, face masks, and eye protection for prevention of covid-19. *The Lancet*, 2020.

[17] Derek K Chu, Elie A Akl, Stephanie Duda, Karla Solo, Sally Yaacoub, Holger J Schünemann, Amena El-harakeh, Antonio Bognanni, Tamara Lotfi, Mark Loeb, et al. Physical distancing, face masks, and eye protection to prevent person-to-person transmission of sars-cov-2 and covid-19: a systematic review and meta-analysis. *The Lancet*, 2020.

[18] Alba Grifoni, Daniela Weiskopf, Sydney I Ramirez, Jose Mateus, Jennifer M Dan, Carolyn Rydyznski Moderbacher, Stephen A Rawlings, Aaron Sutherland, Lakshmanane Premkumar, Ramesh S Jadi, et al. Targets of t cell responses to sars-cov-2 coronavirus in humans with covid-19 disease and unexposed individuals. *Cell*, 2020.

[19] Jose Mateus, Alba Grifoni, Alison Tarke, John Sidney, Sydney I Ramirez, Jennifer M Dan, Zoe C Burger, Stephen A Rawlings, Davey M Smith, Elizabeth Phillips, et al. Selective and cross-reactive sars-cov-2 t cell epitopes in unexposed humans. *Science*, 2020.

[20] Arne Sattler, Stefan Angermair, Helena Stockmann, Katrin Moira Heim, Dmytro Khadzhynov, Sascha Treskatsch, Fabian Halleck, Martin E Kreis, Katja Kotsch, et al. Sars-cov-2 specific t-cell responses and correlations with covid-19 patient predisposition. *The Journal of Clinical Investigation*, 2020.

[21] EISTAT. Primi risultati dell'indagine di sieroprevalenza sul sars-cov-2, August 03,2020. https://www.istat.it/it/archivio/246156.

[22] Xin Xu, Jian Sun, Sheng Nie, Huiyuan Li, Yaozhong Kong, Min Liang, Jinlin Hou, Xianzhong Huang, Dongfeng Li, Tean Ma, et al. Seroprevalence of immunoglobulin m and g antibodies against sars-cov-2 in china. *Nature Medicine*, pages 1–3, 2020.

[23] Fiona P Havers, Carrie Reed, Travis Lim, Joel M Montgomery, John D Klena, Aron J Hall, Alicia M Fry, Deborah L Cannon, Cheng-Feng Chiang, Aridth Gibbons, et al. Seroprevalence of antibodies to sars-cov-2 in 10 sites in the united states, march 23-may 12, 2020. *JAMA internal medicine*, 180(12):1576–1586, 2020.

[24] Luigi Palatella, Fabio Vanni, and David Lambert. A phenomenological estimate of the true scale of covid-19 from primary data. *Chaos, Solitons & Fractals*, page 110854, 2021.

[25] CovidActNow. Covid act now, 2020. https://covidactnow.org.

[26] Seth Flaxman, Swapnil Mishra, Axel Gandy, H Juliette T Unwin, Thomas A Mellan, Helen Coupland, Charles Whittaker, Harrison Zhu, Tresnia Berah, Jeffrey W Eaton, et al. Estimating the effects of non-pharmaceutical interventions on covid-19 in europe. *Nature*, 584(7820):257–261, 2020.

[27] Bette Korber, Will M Fischer, Sandrasegaram Gnanakaran, Hyejin Yoon, James Theiler, Werner Abfalterer, Nick Hengartner, Elena E Giorgi, Tanmoy Bhattacharya, Brian Foley, et al. Tracking changes in sars-cov-2 spike: evidence that d614g increases infectivity of the covid-19 virus. *Cell*, 182(4):812–827, 2020.

[28] Alexandru Dan Corlan and John Ross. Kinetics methods for clinical epidemiology problems. *Proceedings of the National Academy of Sciences*, 112(46):14150–14155, 2015.

[29] J Liu. A rate equation approach to model the denaturation or replication behavior of the sars coronavirus. *Forschung im Ingenieurwesen*, 68(4):227–238, 2004.

[30] Barbara A Jones, Justin Lessler, Simone Bianco, and James H Kaufman. Statistical mechanics and thermodynamics of viral evolution. *PloS one*, 10(9):e0137482, 2015.

[31] Te Faye Yap, Zhen Liu, Rachel A Shveda, and Daniel Preston. A predictive model of the temperature-dependent inactivation of coronaviruses. *Applied Physics Letters*, 117, 08 2020.
